# How to Report Light Exposure in Human Chronobiology and Sleep Research Experiments

**DOI:** 10.3390/clockssleep1030024

**Published:** 2019-06-26

**Authors:** Manuel Spitschan, Oliver Stefani, Peter Blattner, Claude Gronfier, Steven W. Lockley, Robert J. Lucas

**Affiliations:** 1 Department of Experimental Psychology, University of Oxford, Oxford OX2 6GG, UK; 2 Centre for Chronobiology, Psychiatric Hospital of the University of Basel (UPK), 4002 Basel, Switzerland; 3 Transfaculty Research Platform Molecular and Cognitive Neurosciences, University of Basel, 4055 Basel, Switzerland; 4 Federal Institute of Metrology METAS, 3003 Bern-Wabern, Switzerland; 5 Lyon Neuroscience Research Center, Waking team, INSERM UMRS 1028, CNRS UMR 5292, Université Claude Bernard Lyon 1, Université de Lyon, F-69000 Lyon, France; 6 Division of Sleep and Circadian Disorders, Brigham and Women’s Hospital, Boston, MA 02115, USA; 7 Division of Sleep Medicine, Harvard Medical School, Boston, MA 02115, USA; 8 Monash Institute of Cognitive and Clinical Neurosciences, School of Psychological Sciences, Monash University, 18 Innovation Walk, Clayton, VIC 3800, Australia; 9 Division of Neuroscience and Experimental Psychology, Faculty of Biology, Medicine and Health, University of Manchester, Manchester M13 9PT, UK

**Keywords:** chronobiology, sleep research, environmental psychology, light exposure, reporting, retina, non-visual function, reproducible research

## Abstract

Exposure to light has short- and long-term impacts on non-visual responses in humans. While many aspects related to non-visual light sensitivity have been characterised (such as the action spectrum for melatonin suppression), much remains to be elucidated. Here, we provide a set of minimum reporting guidelines for reporting the stimulus conditions involving light as an intervention in chronobiology, sleep research and environmental psychology experiments. Corresponding to the current state-of-the-art knowledge (June 2019), these are (*i*) measure and report the spectral power distribution of the acute stimulus from the observer’s point of view; (*ii*) measure and report the spectral power distribution of the background light environment from the observer’s point of view; (*iii*), make spectra available in tabulated form, (*iv*) report α-opic (ir)radiances and illuminance; (*v*) describe the timing properties of stimulus (duration and pattern); (*vi*) describe the spatial properties of stimulus (spatial arrangement and extent), and (*vii*) report measurement conditions and equipment. We supplement the minimum reporting guidelines with optional reporting suggestions and discuss limitations of the reporting scheme.

## 1. Introduction

In addition to allowing us to see, interact with, and navigate in the environment, light has a profound influence on our physiology and behaviour. Exposure to light, for example, changes the size of the pupil [1,2], acutely suppresses the production of melatonin [3,4,5], shifts the timing of circadian rhythms [6], and modulates alertness, temperature and heart rate [5,7]. These effects are mediated, although not equally, by the five photoreceptor classes in the human retina: the cones, the rods, and the intrinsically photosensitive retinal ganglion cells (*ipRGCs*) that express the photopigment melanopsin [8]. There are three classes of cones differing in their spectral tuning: short-wavelength (S) cones (peak spectral sensitivity 420 nm), medium-wavelength (M) cones (530 nm), and long-wavelength (L) cones (558 nm). Rods have a peak spectral sensitivity near 500 nm and melanopsin peaks at 480 nm [9].

Traditionally, when the effects of light on human physiological, psychological and behavioural responses are described, photometric quantities such as illuminance in lux, or luminance in cd/m^2^ have been used. These quantities are weighted by the sum of L and M cones, and therefore do not uniquely specify the action of light on all retinal photoreceptors in the human eye. Indeed, many lights may have the same illuminance but differ vastly in their colour appearance or melanopsin excitation. In other words: (Il)luminance alone is inappropriate to specify the degree to which a spectrum affects the human photoreceptors.

In this article, we provide a set of minimum reporting guidelines (Box 1) for reporting the properties of light in chronobiology, sleep research and environmental psychology. The purpose of establishing reporting guidelines is three-fold: (*i*) to add insight into possible mechanisms of a discovered effect in a retinally-referenced framework; (*ii*) to allow for replicability of studies by other research groups (current and future), and (*iii*) to allow for inclusion of studies in meta-analyses and systematic reviews. In addition to minimum reporting guidelines, we also discuss optional reporting and open questions. We provide an example in Appendix A.

Box 1 **Minimum reporting guidelines.**

Measure and report the spectral power distribution of the acute stimulus from the observer’s point of view at a known and specified angle and distance from the sourceMeasure and report the spectral power distribution of the background light environment from the observer’s point of view at a known and specified angle and distance from the source Make spectra available in tabulated formReport α-opic (ir)radiances and illuminanceDescribe the timing properties of stimulus (clock time, duration and pattern)Describe the spatial properties of stimulus (spatial arrangement and extent)Report measurement conditions and equipment

***What this article is not****.* This article does not provide a theoretical introduction into designing in-laboratory experiments. The focus is to describe minimum normative guidelines for reporting light exposure in in-laboratory experiments. This article is also not a standard in the regulatory sense, and does not arbitrate between different models of circadian phototransduction (e.g., [10,11]). For readers wishing to expand their knowledge of ancillary topics, we recommend consulting recent reviews on the effects of light on circadian rhythms, sleep and cognition [12,13], colour vision and colorimetry [14,15], lighting and human factors [16], field studies of light exposure [17], and photometry [18]. We have written this article for human chronobiology and sleep research experiments, but similar principles apply for experiments with animal models.

## 2. Minimum Reporting Guidelines

### 2.1. Measure and Report the Spectrum of the Acute Stimulus from the Observer’s Point of View

Typically, studies examining the effects of light in chronobiology and sleep research manipulate an aspect of the acute stimulus, e.g., the spectrum or the intensity. All light-mediated effects proceed from the conversion of photons reaching the retina into neural signals, and therefore the relevant stimulus is the spectral power distribution (SPD) of light reaching the retina. A spectrum specifies the amount of energy (or the number of photons) at a given wavelength band. Given that we cannot measure the light reaching the retina—only the retina can “measure” that—, we can measure the following quantities:

(1) The *spectral irradiance* received at the cornea measured as the vertical spectral irradiance, which incorporates a measurement of light from the entire visual field in the plane of the cornea.
(2) The *spectral radiance* emitted from the emitting surface of a given light source, in a given direction. This can be measured relatively easily for planar surfaces, if they are viewed in the direction of the normal surface (“straight-on”), such as a monitor.

While measurements of spectral irradiance depend on the size of the source, the distance of the observer from the source, and the distribution of the emitted light, the spectral radiance does not (at least if it is a homogenous surface). Where there is only one emitting source with no other light sources in the environment, it is under some circumstances possible to convert from the spectral radiance to the corneal irradiance if distance and spatial extent are known.

Spectra are physical and the most “complete” representations of a light stimulus. Any other representations, such as illuminance (in lux) or chromaticity (in *xy* coordinates) are calculated from a spectrum, or (in the case of photometers and colorimeters) are made by filtering light in front of a sensor. The following light specifications are insufficient and should be avoided: illuminance or luminance only, chromaticity, correlated colour temperature (CCT), peak wavelength only, colour appearance (e.g., “orange”), incident or emitted energy or photon flux (without also specifying spectral composition).

Furthermore, simply giving the make and brand, or the type of light source (e.g., “fluorescent”) does not uniquely specify the in situ spectrum which a human observer is actually exposed to, although this information is important to replicate an experiment. This is because neither (il)luminance nor chromaticity uniquely determine the spectrum. There are infinitely many spectra which yield the same set of (il)luminance and chromaticity values (these are called *metamers*).

Where possible, both spectral irradiance (at the cornea) and spectral radiance of the stimulus (if it is a flat surface that the observers are viewing in the direction of the surface normal, i.e., straight-on) should be measured and reported.

Spectra should be measured at a given moment in time. If a particular experimental design is based on a time-varying stimulus (e.g., daylight exposure or colour tuning), then each of these exposure situations needs to be measured and reported. The key goal is to allow other researchers to recreate the light exposure in all stages of the experiment.

Where it is not possible to directly make spectral measurements (e.g., no measurement equipment available) but where the manufacturer has provided a spectrum, it is preferable to report the manufacturer-tabulated spectrum (with appropriate source attribution) rather than no information at all. In those cases, in situ measurements of at least the photopic illuminance need to be performed and reported as well.

### 2.2. Measure and Report Spectrum of the Background Light Environment from the Observer’s Point of View

In multi-hour or multi-day experiments in a laboratory suite, participants often move about or are not restricted to exposure to a specific acute stimulus. For those conditions, the “background” or “dim” light condition should be characterised as well. Typically, this is done by stating that the light was (e.g., <5 lux) but the same level of detail should be provided for background light as for acute stimuli, as described above. Such background “dim” light is not equivalent to full dark adaptation which may move the retinal sensitivity into a very different state than in a constant “dim” light.

In addition, if the acute stimulus is added to a dim-light environment, e.g., where a screen is used in a situation where there is also dim ambient illumination from another source, that ambient illumination should also be characterised as described above.

### 2.3. Make Spectra Available in Tabulated Form

Spectra should be made available in tabulated form at 1–10 nm spacing (depending on the instrument) between 380 and 780 nm, where possible. The table should be included as data files in the Supplementary Material. Where a journal does not offer the capability of making Supplementary Material available, files can be made available on *FigShare* (https://figshare.org/), the *Open Science Framework* (https://osf.io/) or *GitHub* (https://github.com/). Some institutions also offer repositories for research data. 

To future-proof documentation of stimulus conditions and thereby lengthen the “lifetime” of a piece of scientific work, it is strongly recommended that the files are made available as uncompressed ASCII files (in comma-separated or otherwise delimited file format), to avoid the risk of digital obsolescence of proprietary file formats.

In cases where spectra have been specified in the literature, they have typically been given in graphical form, i.e., as plotted lines. While the underlying data were necessary to produce these plots, the graphs themselves are not sufficient to extract key quantitative information about the light source. This is because it is impossible to estimate, e.g., the luminance of a light source “by eye”. There are methods to extract numerical data from graphical data using tools such as *WebPlotDigitizer* [19], though such an approach inevitably produces unnecessary imprecision [20,21,22].

### 2.4. Report α-opic (ir)radiances and Illuminance

While the spectra represent the most complete representation of stimulus properties, it is very difficult to extract key information from a tabulated spectrum alone in relation to its effects on the human retina. To this end, in addition to being reported in tabulated spectral form, the spectral irradiance and radiance measurements of the stimuli and the ambient environment should be converted into absolute α-opic irradiances (for spectral irradiance) expressed in mW/m^2^ or absolute α-opic radiances (for spectral radiance) expressed in mW/(m^2^ sr). This conversion is performed by weighting the spectrum by the spectral sensitivity of each of the five photoreceptors separately (L, M and S cones, rods, and melanopsin-encoded ipRGCs) and summing up the value across wavelength bands. The spectral sensitivities to be used are those recently standardised by the CIE as CIE S 026/E:2018 [9] (toolbox available at https://doi.org/10.25039/S026.2018.TB). The spectral sensitivities used are the physiologically relevant CIE 10° cone fundamentals [23], the CIE rod fundamental, and a standardised melanopsin spectral sensitivity.

In addition, for orientation, the photopic luminance (in cd/m^2^) (for planar surfaces, in the direction of the surface normal) and the photopic illuminance (lux) should also be given. This is to anchor the stimuli to known quantities and get an intuition for the light level in general.

### 2.5. Describe the Timing Properties of Stimulus

The timing of light exposure needs to be specified, i.e., the duration of a given light exposure phase and the specific temporal pattern (constant light, flicker, pulses, ...). This includes both the time spent in a dim background environment, or in the dark, as well as the timing of the acute stimulus. Where constant light sources are used (i.e., those that do not change properties of the spectrum), it is sufficient to specify relative onset and offset times of the stimulus. In protocols where there is a sequence of light flashes at short (millisecond to second, [24]) or medium (minute to hour, [25]) time scales, flash duration and inter-stimulus interval need to be specified. Where dynamic stimuli are used (e.g., movies [26] or screens [27]), minimum reporting should include timing properties of the device (e.g., refresh rate) and the mean properties of the light.

### 2.6. Describe the Spatial Properties of Stimulus

The irradiance arriving at the cornea depends not only on the radiance emitted by the light source, but also on the size of the source and the receiver’s distance to it. Therefore, while radiance and luminance measurements are typically distance-independent, that is not the case for irradiance and illuminance. Therefore, the shape and size of the source (horizontal and vertical), and the observer’s distance and view angle need to be specified. 

*Conversion to visual angle.* The visual angle of an object is the size of the object as a retinal image, i.e., projected onto the retina. The visual angle in degrees *θ* that an object projects onto the retina can be calculated from the object size *S* and the object distance *d* using the formula $\theta =2\mathrm{atan}\frac{0.5S}{d}$.

### 2.7. Report Measurement Conditions

The measurement conditions under which the stimuli were characterised should be given, along with the wavelength sampling, the spectral bandwidth and reporting range of the measurement instrument. The measurement instrument should be specified in terms of sensor, brand and make.

## 3. Optional Reporting

### 3.1. Measure or Estimate Pupil Size of the Study Participant

Where feasible, pupil size of the observer should be measured, in particular when it is assumed that two conditions differ in stimulus properties. When it is impossible or impractical to measure pupil size, it is possible to predict pupil size using the formula for light-adapted pupil size by Watson and Yellott [28], which takes into account field size, luminance, age, and whether or not stimulus is viewed with one or two eyes. While this model ignores non-luminance contributions to pupil control, and assumes a systematic effect of age on crystalline lens density [29,30,31], the estimated pupil size should nonetheless still be reasonably accurate. In experiments where pharmacological pupil dilation has been used, the diameter of the artificial pupil should be reported along with the details of the pharmacological agent used.

### 3.2. Measure or Estimate Percentage Eye Open

It should be noted that most commercially available irradiance/illuminance measuring instruments measure light arriving from ±90°; hence their field of view is a 180° hemisphere. This is not the case for the eye. Although the horizontal field of view with a fixed head and a fixed gaze is 180°, the vertical field of view is at most 130°, decreasing with increasing luminance. At high luminances, the field of view is limited by the upper eyelid (and by the lower eyelid with high luminances from below the horizon) [32]. By measuring irradiance/illuminance at the cornea alone, retinal illuminance cannot be estimated. The estimation or measurement of % eyes open can help in estimating the retinal image area illuminated by a light source. 

### 3.3. Photography or Sketch of Laboratory Arrangement

A (wide-angle) photograph or a sketch of the experimental setting is a simple way of documenting the lighting environment. It helps the reader to understand the environmental conditions of the experiment.

### 3.4. Light Safety Calculations >10,000 cd/m^2^

There are two main mechanisms of possible light-induced damage: (*i*) The heat at which the radiant energy is absorbed in a certain volume causes a temperature rise in the tissue and consequently photothermal damage can occur; (*ii*) Photochemical damage can occur when light is absorbed by molecules and an electrically excited state of these molecules is formed, which in turn can cause chemical changes in the molecules. Due to the low energy of commonly used domestic light levels, heat-induced damage does not usually play a role [33]. The IEC 62471 standard covers all photobiological hazards, thermal and photochemical, which may affect the eye and skin. This standard divides luminaires into risk groups according to their radiant power. The limit value of the blue light-weighted radiance for the photochemical hazard of the retina in risk group 0 (no risk) is 100 W/(m^2^ sr) in the visible wavelength range at an exposure of more than 10,000 s. IEC62471 recommends that detailed measurements for safety reasons are typically not required for sources having a luminance of less than 10,000 cd/m^2^. Compliance with light safety regulations needs to be checked and confirmed on a case-by-case basis.

### 3.5. Radiometric vs. Photon System

Light can not only be defined by energy units (radiometric system) but also be expressed as photons or quanta (photon system) [34]. Photon irradiance (specified in quanta/cm^2^/s or photons/cm^2^/s or a base-10 logarithmic transformation of it, i.e., log_10_ quanta/cm^2^/s or photons/cm^2^/s) describes how many photons arrive at a given detector surface area per second. Photon irradiance can be calculated from the spectral distributions, where available. 

### 3.6. Calculate Age-Dependent Effects on Pre-Receptoral Filtering

Where the examined participant population does not fall near the standard observer age (32 years), it is important to consider the relative reduction of short-wavelength light due to lens yellowing with increased age [31]. The CIE S 026 standard provides a formula for this [9], but it should be noted that it provides an estimate of lens density based on group average and that real density may be different [29,30,31]. It is recommended that stimuli which heavily rely on specific assumed lens properties (e.g., metameric lights) are subjected to a “sensitivity analysis” in which the α-opic irradiance is calculated for a set of candidate observers spanning the age range [35,36].

### 3.7. Reporting Retinal Intensity

Spectral radiance and corneal irradiance are quantities which meaningfully characterise the spectrum of light in the physical world. As the effects of light on the human circadian system are mediated by the retina, however, the quantity that determines biological responses is retinal intensity. This can be calculated from the spectral radiance by multiplying with the pupil area. For luminance, that quantity is given called the troland [37]. Many parameters of the human visual system, e.g., the rod saturation threshold [38], have classically been given as trolands and therefore it remains a useful unit (see CIE S 026 standard [9], footnote on page 10).

## 4. Challenges

The proposed minimum guidelines here are presented as the best-practice method corresponding to the state-of-the-art knowledge. Additional suggestions reflecting further developments will be available as Supplementary Information at doi.org/10.17605/OSF.IO/K3V2T. There are several challenges that any reporting guideline faces, however. We address these here for completeness.

*Field of view.* The vertical irradiance (or illuminance) measured at the cornea does not take into account that facial features may limit the amount of actual retinal illuminance for extended sources [39]. This might lead to an overestimation of actual retinal exposure to light. CIE S 026 recommends a limitation of the field of view in the vertical direction: Depending on the lighting condition, the limitation is 50°–55° above the line-of-sight under low illumination or as low as ~15° under very bright illumination conditions (such as outdoor environments) [9].

*Effect of eye, head and trunk movements.* Under conditions where fixation is not enforced or encouraged by the experimental paradigm, eye, head and trunk movements displace the retinal image. Any stationary physical measurement of irradiance or radiance does not represent this. One strategy to quantify instantaneous light exposure would be to measure eye movements, or use wearable miniature measurement devices worn at eye level.

*Retinal heterogeneities and individual differences.* The spectral sensitivities from the CIE S 026 standard represent a *standard observer* based on average estimates of the cone and rods’ spectral sensitivities obtained from psychophysical data. In addition to the age adjustment parameters, which modifies the lens density function, there are other individual differences affecting the spectral sensitivities [40]. These are not captured by the standard observer. Furthermore, the standard assumes a 10° field size, though we currently do not know the precise contributions of foveal and peripheral regions to circadian photoentrainment.

## 5. Conclusions

The minimum and optional reporting guidelines given in this article provide a reference framework for specifying light exposure and stimulus conditions in chronobiology, sleep research and environmental psychology experiments. We hope that they provide a useful best-practice standard and “recipe” for reporting stimulus conditions. Adhering to the reporting guidelines is, however, primarily the responsibility of the authors of a given scientific article. This responsibility is shared with peer reviewers, who we invite to prompt the authors to make spectral data available, and journal editors, who we invite to institute and enforce reporting guidelines in their respective journals. In the long run, this approach will enable meta-analyses of the data and allow researchers to replicate protocols and studies.

## Supplementary Materials

Supplementary materials can be found at https://osf.io/k3v2t/.

## Appendix A. Example Reporting

### Narrative Description

In this experiment, participants were exposed to <8 lux environmental light between 18:00 and 22:00 and to 2 h of experimental light between 22:00 and 00:00 (EL_1_). The environmental light was provided by fluorescent “white” room lighting. Participants were either sitting at the table (DL_1_, ~2 lux, ~10 mW/m^2^ total irradiance measured in the vertical plane at eye level) or on the sofa (DL_2;_ ~4 lux, ~17 mW/m^2^ total irradiance measured in the vertical plane at eye level). During the 2 h experimental light exposure, participants were exposed to an acute stimulus (~175 lux; 581 mW/m^2^ measured in the vertical plane at eye level). The experimental light stimulus was a homogeneous annular surface viewed at 20 cm distance (inner diameter = 12°, outer diameter = 40°). The inner and outer regions outside of the surface were not illuminated. A small dim red fixation cross was provided which subjects fixated on. The experimental light was constant during the exposure phase (2 h). Spectral measurements (range 380–780 nm; optical resolution of device 4.5 nm; reported wavelength sampling 1 nm) were performed with JETI spectroval 1501 (JETI Technische Instrumente GmbH, Jena, Germany). Illuminance, α-opic (ir)radiances and spectral irradiances of all conditions are given in Table A1.

**Table A1:** Stimulus specification.

*Condition*	DL_1_	DL_2_	EL_1_
Illuminance [lux]	2.41	4.16	173.50
S-cone-opic irradiance (mW/m^2^)	0.62	1.12	1.630
M-cone-opic irradiance (mW/m^2^)	2.75	4.74	133.78
L-cone-opic irradiance (mW/m^2^)	3.98	6.86	313.12
Rhodopic irradiance (mW/m^2^)	1.73	2.97	80.23
Melanopic irradiance (mW/m^2^)	1.27	2.17	52.99
**Wavelength [nm]**	**Spectral irradiance [W/(m^2^ nm)]**		
380	0.00E+00	0.00E+00	0.00E+00
381	0.00E+00	0.00E+00	3.54E-06
382	0.00E+00	0.00E+00	6.94E-06
383	0.00E+00	0.00E+00	1.07E-05
⋮	⋮	⋮	⋮
777	5.53E-06	4.55E-06	1.07E-05
778	6.20E-06	4.80E-06	1.55E-05
779	6.37E-06	5.94E-06	1.29E-05
780	6.75E-06	6.86E-06	8.15E-06

*Note:* Full spectrum not given due to space constraints in this example. Tabulated spectra should be made available as Supplementary Material (as comma-separated file, CSV).
